# Retrospective evaluation of choroidal and retinal changes in patients with diabetic peripheral neuropathy but no diabetic retinopathy

**DOI:** 10.7717/peerj.20662

**Published:** 2026-03-16

**Authors:** Raşit Dilek

**Affiliations:** Ophthalmology Department, Konya City Hospital, Konya, Turkey

**Keywords:** Diabetic peripheral neuropathy, Choroidal vascular index, Diabetic retinopathy.

## Abstract

**Purpose:**

To compare macular and choroidal findings in patients with diabetic peripheral neuropathy but no diabetic retinopathy with healthy patients.

**Method:**

We compared the ganglion cell-inner plexiform layer (GCIPL), retinal nerve fiber layer (RNFL), central macular thickness (CMT), subfoveal choroidal thickness (SFCT) and choroidal vascular index (CVI) measurements in patients with diabetic peripheral neuropathy (DPN) without diabetic retinopathy (DR) with healthy individuals.

**Results:**

Mean CMT, RNFL and SFCT values were compared in the study and control groups. There was no statistically significant difference between the groups (*p* = 0.139, 0.372, and 0.594, respectively). The mean, minimum, superonasal, superior, superotemporal, inferotemporal, inferior and inferonasal GCIPL values were compared in the study group. Only the inferior and inferonasal sectors showed a significant difference (*p* = 0.012 and 0.023, respectively), while no difference was found in the other sectors (*p* > 0.05). Mean stromal area (SA), luminal area (LA) and total choroidal area (TCA) values were compared in the study and control groups. While a significant difference was found between the study and control groups in LA and TCA (*p* = 0.009 and *p* = 0.008, respectively), no significant difference was found in SA (*p* = 0.121). The CVI was 0.68850 in the study group and 0.68575 in the control group, and no significant difference was found between the groups (*p* = 0.456).

**Conclusion:**

The results obtained demonstrated a statistically significant increase in LA and TCA in the study group, although CVI remained constant. Furthermore, a statistically significant decrease in GCIPL thickness in the inferior and inferonasal sectors of the study group was identified.

## Introduction

Diabetes mellitus (DM) is a chronic systemic disease associated with significant morbidity and mortality due to its multi-organ complications. These complications are broadly classified into vascular (*e.g*., retinopathy, nephropathy, neuropathy, coronary artery disease) and non-vascular (*e.g*., infections, skin changes, cognitive decline) types (Saini, Kochar & Poonia, 2021).

Diabetic polyneuropathy (DPN), a common long-term microvascular complication of DM, affects sensory, motor, and autonomic nerves, leading to disability, foot ulcers, and increased cardiovascular risk (Boulton et al., 2004). Its prevalence ranges from 7% in the first year of diagnosis to over 50% after 25 years, and even higher when subclinical cases are included (Schmader, 2002). Current diagnostic methods—clinical examination, nerve conduction studies, and biopsies—are either invasive or subjective, highlighting the need for objective, non-invasive biomarkers (Feldman et al., 2019; Selvarajah et al., 2019).

The retina, uniquely accessible for *in vivo* assessment, is rich in sensory neurons and may reflect early neurodegenerative changes in DPN (Kern & Barber, 2008). Studies have shown that DM can lead to the loss of ganglion cells (GCs) and thinning of the retinal nerve fibre layer (RNFL), potentially even before microvascular damage is apparent (Bailey & Sparrow, 2001; Lopes de Faria et al., 2001). Optical coherence tomography (OCT) now allows precise measurement of parameters like ganglion cell–inner plexiform layer (GCIPL) and RNFL thickness, which may serve as indicators of retinal neurodegeneration (Shahidi et al., 2012).

Additionally, the choroid, responsible for outer retinal blood supply, undergoes diabetes-related microvascular changes (Venturini et al., 2006). While choroidal thickness (CT) has been used to study these changes, it is influenced by systemic and ocular factors (Lovasik & Kergoat, 1993; Riva et al., 1997). The choroidal vascularity index (CVI), derived using a binarisation technique, offers a more stable and reproducible alternative (Abdolrahimzadeh et al., 2023; Tan et al., 2012).

The purpose of this study is to compare GCIPL, RNFL, central macular thickness (CMT), CT, and CVI between patients with DPN (without DR) and healthy controls to evaluate the potential of GCIPL and CVI as non-invasive biomarkers for DPN. Considering that DPN reflects systemic neurodegenerative processes, we hypothesized that neuronal axonal damage may occur in the retina even before the onset of clinically detectable DR. Therefore, we expected to observe a decrease in the GCIPL thickness and CVI in these patients. We hypothesised that in individuals with DPN, GCIPL thickness and CVI are significantly lower compared to healthy individuals.

## Materials and Methods

Ethics committee approval was obtained from the Necmettin Erbakan University Faculty of Medicine Ethics Committee for this retrospective study (Ethical appoval number: 2024/5366). This study was performed in accordance with the principles of the Declaration of Helsinki. Written informed consent forms were received from all participants.

Patients who were admitted to Konya City Hospital between January 2022 and December 2024, did not have DR on routine ophthalmological examination, were taking medication for DPN (duloxetine, amitriptyline, pregabalin, gabapentin, alpha-lipoic acid, and opioid or opioid-like medications *e.g*., tramadol), and had electromyography (EMG) reports in the system were retrospectively reviewed. Patients were included in the study if they had EMG reports suggestive of DPN, were taking medication for DPN, had ophthalmic examination records without findings suggestive of DR, and had macular cube, optic disc, and Edi-OCT measurements on the OCT device. Patients with any other neurological disease (Parkinson’s disease, Alzheimer’s disease, cerebrovascular disease, *etc*.), and other concomitant ocular diseases that might affect the OCT measurement (cataract, glaucoma, macular disease, previous vitreoretinal surgery, or previous optic neuropathy, *etc*.) were excluded. Patients with spherical equivalent (SE) greater than ±6 diopters and astigmatism greater than ±3 diopters were also excluded. The control group consisted of age- and sex-matched healthy subjects without any ocular or systemic disease. Patients with SE greater than ±6 diopters and astigmatism greater than ±3 diopters were also excluded from the control group.

A total of 110 eyes of 110 patients were included in the study group and 110 eyes of 110 subjects were included in the control group. In both the patient and control groups, the right eye of the patient was included in the study.

**Figure 1:**
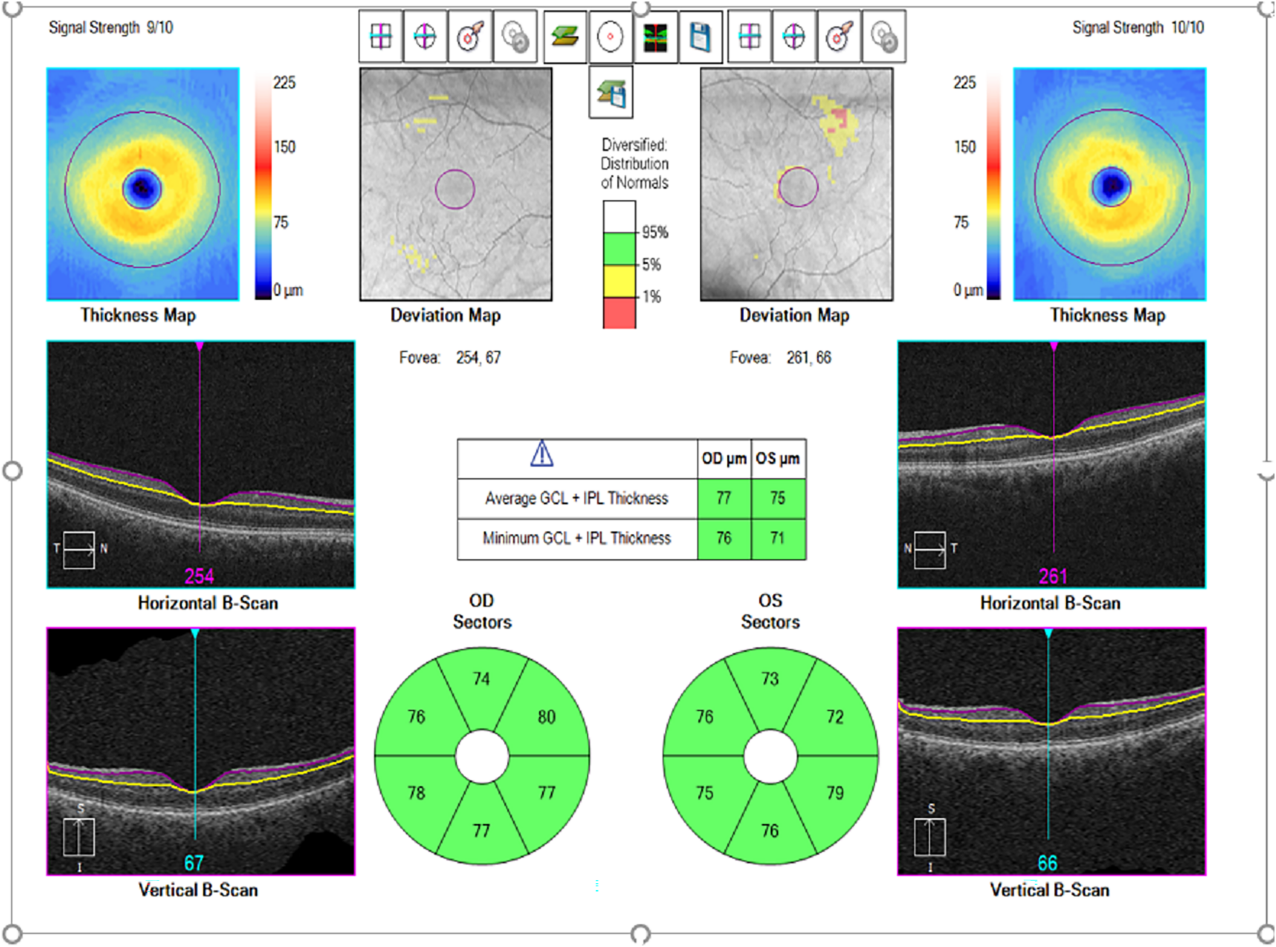
Example of macular ganglion cell inner plexiform layer (GCIPL) thickness measurement automatically determined by optical coherence tomography. Macular GCIPL thickness maps.

Macular GC analysis (GCA) of Cirrus high-resolution OCT (Cirrus HD-OCT, model 5,000; Carl Zeiss Meditec) measures GCIPL thickness within an elliptical ring around the fovea. As the GCIPL represents retinal ganglion cell bodies and dendrites, this analysis is expected to effectively detect structural abnormalities in the macular area. GCIPL and CMT were measured in all patients by the same experienced technician. The Macular Cube 512 × 128 scan mode was used to scan a 6 × 6 mm area centred on the fovea. CMT was automatically calculated during analysis of a 1.0 μm retinal map by rapid macular scanning. Software version 6.0 of the GCA algorithm (Carl Zeiss Meditec) was used to process the data and measure macular GCIPL thickness within a 6 × 6 × 2 mm cube centred on the fovea. Mean, minimum, and sectoral (superonasal, superior, superotemporal, inferotemporal, inferior, and inferonasal) macular GCIPL thickness measurements were analysed (Fig. 1). Only complete, well-centred, high-quality scans were used for these measurements. RNFL thickness was measured by scanning the peripapillary circle of approximately 3.46 mm diameter with the eye tracking system active, using the Optic Disc Cube 200 × 200 protocol. Global and regional (temporal, superotemporal, superonasal, nasal, inferonasal and inferotemporal sectors) RNFL thicknesses were obtained as a result of the analysis and the global measurement was taken as the mean RNFL thickness (Brautaset et al., 2016). For choroidal imaging, a high-resolution 21-line EDI foveal scan was obtained from each participant. Images with poor signal strength or focal signal loss or motion artefacts were not used. Subfoveal choroidal thickness (SFCT) was defined as the distance between the outer edge of the hyperreflective RPE and the inner surface of the sclera and was measured manually from the fovea by the same experienced independent masked observer using the same instrument software (Yiu et al., 2014). This measurement was repeated three times consecutively and the mean was recorded. OCT measurements of all participants were performed between 08:00 and 12:00 h to avoid diurnal variations and were included in the study.

**Figure 2:**
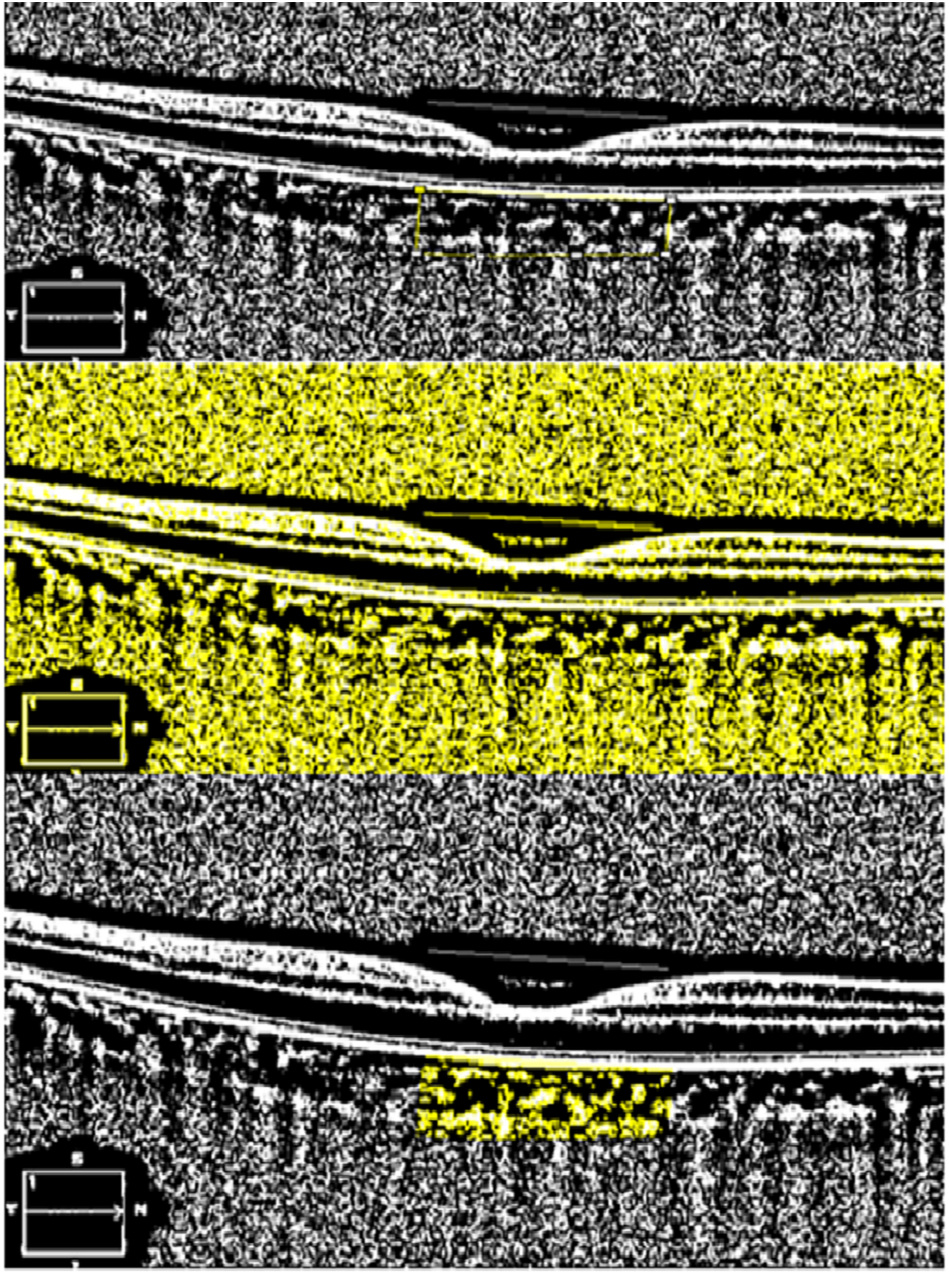
Calculation of choroidal vascular index with ImageJ. Determining the area to be calculated, applying Niblack’s auto local threshold and determining the regions representing the lumen area using the color threshold tool.

ImageJ software (https://imagej.net; version 1.53; National Institutes of Health, Bethesda, MD, USA) was used for CVI analysis. This calculation was performed according to the method previously described by Agrawal et al. (2016). Each EDI-OCT image was opened in ImageJ software for calculation. The subfoveal area from the RPE to the choroidoscleral junction was marked with the polygon selection tool 750 μm nasal and 750 μm temporal to the fovea. The marked image of the total choroidal area (TCA) was then binarised using the Niblack autolocal thresholding method. Specifically, the image was converted to 8 bits and an automatic local threshold was applied. The luminal area (LA) was highlighted by converting the image into a red-green-blue colour space and then applying a colour threshold. The first value automatically calculated by the program represented the TCA, while the second value represented the LA. The stromal area (SA) was calculated by subtracting the LA from the TCA. The CVI is calculated by dividing the LA by the TCA. The CVI was calculated by another investigator (RD) independent of the patients’ diagnoses, and the average of each calculated parameter was used for analysis (Fig. 2).

Statistical analysis was performed using SPSS software (version 22; SPSS Inc., Chicago, IL, USA). The Kolmogorov-Smirnov test was used to determine whether the data were normally distributed. Parametric or non-parametric tests were preferred depending on whether the data showed a normal distribution or not. Independent samples t-test, one of the parametric tests, and Mann-Whitney U test, one of the non-parametric tests, were used to compare parameters between groups. *P* value of less than 0.05 was considered statistically significant. The intraclass correlation coefficient (ICC) was used to assess intraobserver reliability for SFCT, LA, SA, and TCA measurements. The primary endpoint of this study was defined as the mean GCIPL thickness. Analysis was performed at the patient level, including only the right eye of each participant, ensuring independence of observations. The observed mean GCIPL thickness was 76.39 ± 14.01 µm in the study group and 76.66 ± 9.24 µm in the control group, with an observed difference of 0.27 µm. The pooled standard deviation was calculated as 11.86 µm, yielding an observed effect size (Cohen’s d) of 0.023, indicating a very small effect. Based on a two-sided t-test with α = 0.05 and *n* = 110 per group, the minimum detectable difference (MDD) with 80% power is approximately 4.46 µm. Because the observed difference is much smaller than this threshold, the study is underpowered to detect the observed GCIPL difference. Therefore, the power analysis is best interpreted as a *post-hoc* assessment reflecting the negligible intergroup difference, rather than a prospective sample size calculation.

## Results

The mean age of the study group, which comprised 54 males and 56 females with DPN and no DR, was 61.5 years, while the mean age of the control group, which comprised 53 males and 57 females, was 63.2 years. A statistical analysis revealed no significant difference between the study group and the control group with regard to age or gender (*p* = 0.143 and 0.887, respectively) (Table 1). In addition, 54 of 110 patients were on antihypertensive medication due to hypertension (HT), and there was no statistically significant difference in age between hypertensive and non-hypertensive patients (*p* = 0.720).

**Table 1:** Gender, age, best corrected visual acuity (BCVA), mean refractive error, intraoculer pressure values.

Parameter	Study group (*n*:110)	Control group (*n*:110)	*P* value
Gender (male/female ratio)	54 (%49)/56 (%51)	53 (%48)/57 (%52)	0.887
Age (years)	61.5 (±10.58)	63.2 (±9.17)	0.143
Mean BCVA (Snellen chart)	0.92 (±0.12)	0.93 (±0.08)	0.231
Mean refractive error (diopter)	−1.98 (±1.31)	−1.56 (±1.02)	0.078
Intraocular pressure (mmHg)	15.21 (±2.25)	14.92 (±2.12)	0.321
Systolic blood pressure	132.09 (±16.76)	116.45 (±5.08)	<0.001
Diastolic blood pressure	81.50 (±111.72)	74.33 (±6.01)	<0.001

The study group had mean CMT, RNFL, and SFCT values of 236.69, 94.51 and 319.04 µm, respectively, while the control group had values of 243.16, 95.73, and 316.71 µm. There was no statistically significant difference between the groups (*p* = 0.139, 0.372, and 0.594, respectively) (Table 2). Furthermore, there was no statistically significant difference between patients taking antihypertensive medication and those not taking antihypertensive medication (*p* > 0.05) (Table 3).

**Table 2:** Mean central macular thickness (CMK), retinal nerve fibre layer (RNFL), subfoveal chooroidal thickness (SFCT), ganglion cell-inner pleksiform layer (GCIPL) thickness, stromal area (SA), luminal area (LA), total choroidal area (TCA), choroidal vascular index (CVI) values.

Parameter	Study group	Control group	*P* value
Mean CMT thickness	236.69 (±29.51) µm	243.16 (±32.04) µm	0.139
Mean RNFL thickness	94.51 (±10.62) µm	95.73 (±9.15) µm	0.372
Mean SFCT thickness	319.04 (±32.78) µm	316.71 (±30.93) µm	0.594
GCIPL thickness			
• Mean	76.39 (±14.01) µm	76.66 (±9.24) µm	0.868
• Minimum	71.27 (±10.61) µm	71.42 (±7.53) µm	0.852
• Superonasal	79.56 (±15.75) µm	80.07 (±12.39) µm	0.797
• Superior	79.12 (±15.28) µm	79.58 (±11.97) µm	0.902
• Superotemporal	76.27 (±13.92) µm	77.01 (±9.78) µm	0.854
• Inferiotemporal	72.64 (±11.36) µm	73.04 (±8.03) µm	0.841
• Inferior	70.70 (±10.09) µm	73.36 (±8.22) µm	**0.012**
• Inferionasal	73.48 (±10.67) µm	76.01 (±9.07) µm	**0.023**
Stromal area (SA) (µm^2^)	306,719 (±42,082) µm^2^	299,264 (±41,749) µm^2^	0.121
Luminal area (LA) (µm^2^l)	677,666 (±69,463) µm^2^	653,737 (±71,431) µm^2^	**0.009**
Total choroidal area (TCA) (µm^2^)	984,386 (±99,180) µm^2^	953,001 (±91,441) µm^2^	**0.008**
Choroidal vascular index (CVI)	0.68850 (±0.020988)	0.68575 (±0.034046)	0.456

**Note:**

Values in bold are statistically significant.

**Table 3:** Mean central macular thickness (CMT), retinal nerve fiber layer (RNFL), subfoveal choroidal thickness (SFCT), ganglion cell-inner plexiform layer (GCIPL) thickness, stromal area (SA), luminal area (LA), total choroidal area (TCA), choroidal vascular index (CVI) values in hypertensive and non-hypertensive groups.

Parameter	Study group (with HT, *n*:54)	Study group (with no HT, *n*:56)	*P* value
Mean CMT thickness	232.81 (±27.28)	240.43 (±31.31)	0.177
Mean RNFL thickness	93.85 (±10.27)	95.14 (±11.00)	0.526
Mean SFCT thickness	317.04 (±32.37)	320.36 (±33.35)	0.532
GCIPL thickness			
• Mean	75.85 (±15.98)	76.91 (±11.90)	0.694
• Minimum	71.07 (±11.21) µm	71.55 (±8.99) µm	0.725
• Superonasal	79.27 (±14.25) µm	79.85 (±11.58) µm	0.456
• Superior	78.85 (±15.28) µm	79.02 (±10.96) µm	0.889
• Superotemporal	75.57 (±14.56) µm	76.09 (±9.82) µm	0.721
• Inferiotemporal	72.33 (±10.44) µm	72.88 (±9.93) µm	0.715
• Inferior	70.38 (±10.89) µm	70.72 (±10.52) µm	0.689
• Inferionasal	73.86 (±10.67) µm	73.01 (±10.92) µm	0.521
Stromal area (SA) (µm^2^)	305,918 (±35,449) µm^2^	307491 (±39,671) µm^2^	0.827
Luminal area (LA) (µm^2^)	678,872 (±65,518) µm^2^	676504 (±76,315) µm^2^	0.862
Total choroidal area (TCA) (µm^2^)	984,791 (±92,185) µm^2^	983996 (±106,325) µm^2^	0.967
Choroidal vascular index (CVI)	0.68,950 (±0.19812)	0.68755 (±0.22200)	0.628
Age (years)	61.13 (±10.35) year	61.86 (±10.88) year	0.720

**Figure 3:**
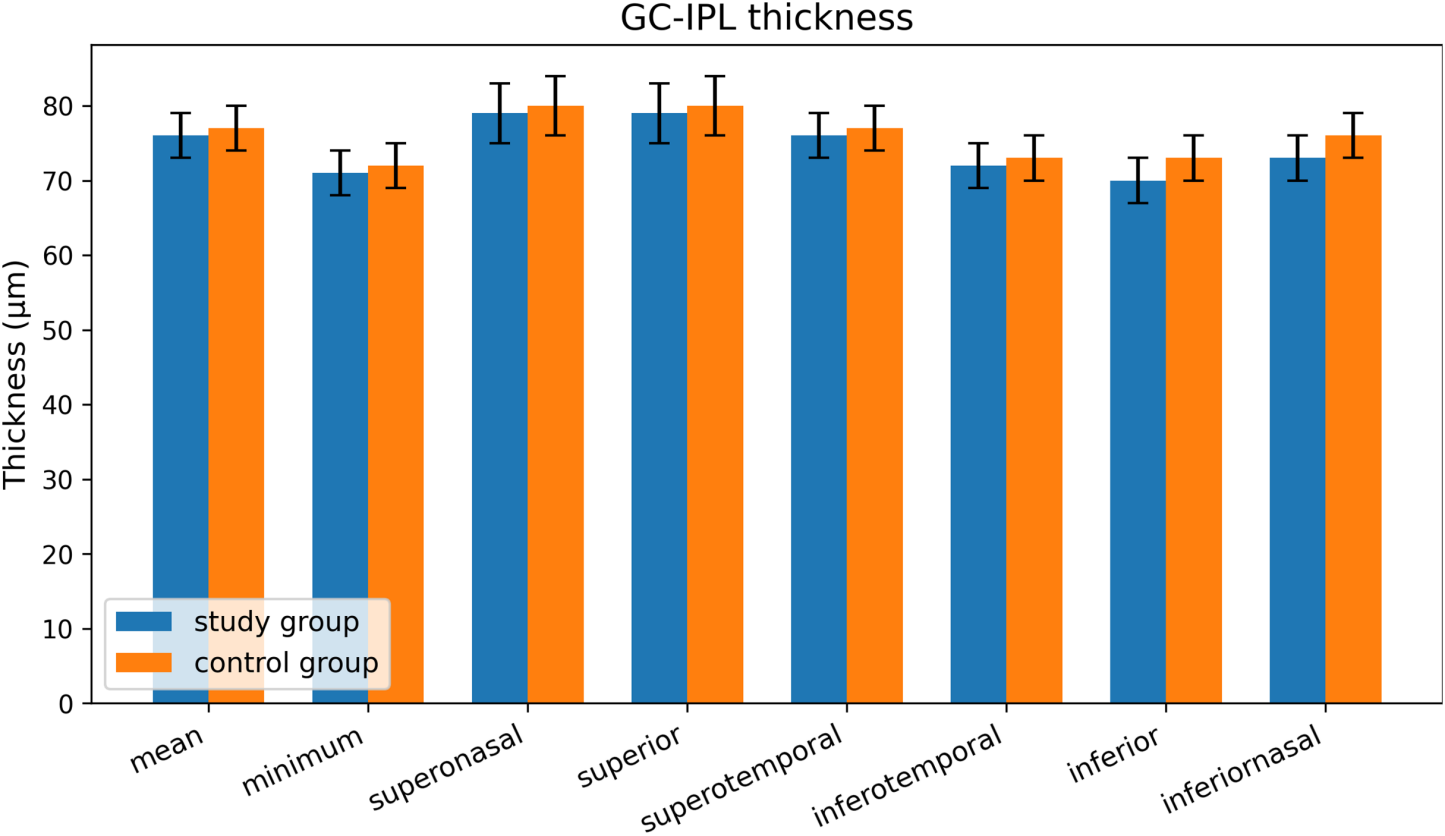
Sectorial GCIPLchangies.

In the study group, the mean, minimum, superonasal, superior, superotemporal, inferotemporal, inferior, and inferonasal GCIPL values were 76.39, 71.27, 79.56, 79.12, 76.27, 72.64, 70.70 and 73.48 µm, respectively. The values for the control group were 76.66, 71.42, 80.07, 79.58, 77.01, 73.04, 73.36 and 76.01 µm, respectively (Fig. 3). When these values were compared between the two groups, only the inferior and inferonasal sectors showed a significant difference (*p* = 0.012 and 0.023, respectively), while no difference was found in the other sectors (*p* > 0.05) (Table 2). Furthermore, there was no significant difference between patients who received HT medication and those who did not (*p* > 0.05) (Table 3).

The mean SA, LA and TCA in the study group were 306.719, 677.666 and 984.386 µm^2^, respectively, whereas in the control group, these values were 299.264, 653.737 and 953.001 µm^2^, respectively. While a significant difference was found between the study and control groups in LA and TCA (*p* = 0.009 and *p* = 0.008, respectively), no significant difference was found in SA (*p* = 0.121). The CVI was 0.68850 in the study group and 0.68575 in the control group, and no significant difference was found between the groups (*p* = 0.456), (Table 2). Furthermore, there was no statistically significant difference between patients taking antihypertensive medication and those not taking antihypertensive medication (*p* > 0.05), (Table 3).

ICC values for SFCT, SA, LA and TCA measurements were greater than 70% with a 95% confidence interval in both control and study groups and *p* = 0.000.

## Discussion

The choroid is responsible for supplying blood to the outer retinal layers and any disruption to its structure or vascularisation can have a detrimental effect on retinal function. A number of studies in the literature have used SFCT as a marker to assess the health of the choroid in patients with DM and other retinal disorders. Changes in the choroidal vascular structure of patients with DM have been defined as increased tortuosity, enlargement and narrowing, hypercellularity, microaneurysm formation, loss of choriocapillaries and sinus-like structure formation between choroidal lobules (Cao et al., 1998). In the literature, studies on SFCT in patients with DM have reported different results, such as thinning, constant, or thickening of SFCT. Kim et al. (2013) found that SFCT increased with the severity of DR. However, Sudhalkar et al. (2015) found choroidal thinning with increasing severity of DR. Similar findings regarding a decrease in SFCT in patients with DR were observed in studies by Vujosevic et al. (2012), Marques (2016), Galgauskas et al. (2016) and Lee, Lim & Shin (2013). On the other hand, some studies reported no significant difference between patients with DM and healthy groups in terms of SFCT (Lee, Lim & Shin, 2013; Tan et al., 2016; Wang & Tao, 2019). The conflicting results seen in various studies suggest that SFCT is not well correlated with the presence of DR or diabetic macular oedema (DME). We speculate that this poor correlation may be due to the fact that SFCT is influenced by a number of systemic and ocular variables such as age, axial length, intraocular pressure and blood pressure. In the present study, no significant difference was found in SFCT between the patient group and the control group. In addition, although Gupta et al. (2018) reported that patients with HT had significantly thinner SFCT compared to patients without HT, no significant difference was also found between patients taking medication for HT and those not taking medication for HT in our study.

In our study, we also used CVI as an additional comparison tool to SFCT to understand the structural changes in the choroid in the group with DPN and no DR (study group). CVI is a novel marker that represents the proportion of the choroidal vasculature, including both large choroidal vessels and the choriocapillaris. Gupta et al. (2018) reported a statistically significant decrease in CVI in patients with DR and DME compared with the healthy control group and also demonstrated that there was a decrease in CVI with increasing severity of DR. In another study, Sidorczuk et al. (2022) reported that patients with DM had lower SFCT and CVI than healthy controls, but did not report a significant difference in LA, SA, and TCA. Kim et al. (2018) reported a decrease in CVI in patients with type 2 DM regardless of DR stage. In particular, they reported a lower mean CVI in the proliferative DR group compared to healthy controls, non-DR and mild/moderate nonproliferative DR groups. As reported in the mentioned studies, it can be stated that there is a general decrease in CVI with increasing severity of DR. Various studies reported that this was related to increased loss of choriocapillaris in diabetic patients compared to non-diabetic patients (Cao et al., 1998). However, the present study revealed no significant alterations in CVI. This may be attributed to the absence of choriocapillaris loss, given that the study group comprised patients who had not yet developed DR. Moreover, the constancy of CVI may be attributable to the rise in LA and consequently, TCA, resulting from the dilation of choroidal vessels due to autonomic dysregulation triggered by DPN.

Studies utilizing swept-source OCTA have detected greater flow deficits in the choriocapillaris of diabetic individuals without retinopathy compared to healthy controls. These studies evaluated flow deficits ranging from 200 to 800 μm^2^ and demonstrated that deficits larger than 600 μm² provided the highest diagnostic accuracy for identifying diabetic changes (AUC: 0.912). In contrast, analyzing all flow deficits collectively resulted in a marked reduction in diagnostic accuracy (AUC: 0.579) (Chua et al., 2024). Similarly, the increase in luminal area observed in our study produced an effect comparable to that seen with swept-source OCTA and supports its potential utility as a reliable biomarker.

Since the choroid is mostly under the control of the autonomic nervous system and has an extremely rich neuronal innervation from intrinsic choroidal neurons, it can be considered one of the first tissues to be affected in patients with DPN (Low et al., 2004). Furthermore, Tan et al. (2016) reported an increase in SCFT with a statistically significant decrease in CVI in the DM group and a corresponding increase in TCA in the eyes of patients with DR. In another study, Borrelli et al. (2020) investigated the relationship between foveal choriocapillary perfusion and LA, SA and CVI in healthy subjects. They reported that although initial choroidal enlargement was associated with an increase in choriocapillary perfusion, further increases in LA and SA appeared to be associated with a progressive decrease in choriocapillary perfusion and that initial choroidal thickening in early DR may ultimately compromise choriocapillary perfusion and lead to choroidal thinning. In addition, higher CVI was associated with lower central retinal thickness and higher SFCT in the study by Kim et al. (2018). Furthermore, Gupta et al. (2018) reported that hypertensive patients had thinner choroid compared to non-hypertensive patients in the DM group, but CVI was not affected by HT. Similarly, in the present study, no significant difference in terms of CVI was observed between the group treated with antihypertensive medication and the group not treated with antihypertensive medication.

Previous studies have reported inconsistent results regarding the effect of DM or hyperglycaemia on GCIPL. In one study, an increase in GCIPL thickness was observed in diabetic patients without proliferative DR (Gerendas et al., 2020). Gundogan et al. (2016) found that GCC was thinner in patients with type 1 DM without clinically diagnosed DR compared to controls, whereas Lima et al. (2010) found that GCC was thinner in patients with type 2 diabetes compared to controls without DM. Bloch et al. (2017) reported that GCC thickness was not associated with blood glucose level or DM. Srinivasan et al. (2016) reported a more significant loss of GCC in patients with DPN than in patients with DM but without DPN. Hegazy et al. (2017) compared the GCC thickness of patients with diabetes and healthy controls and found no statistically significant difference between the study and control groups in terms of age, gender, BCVA, IOP, C/D ratio or SFCT. Another study by Van Dijk et al. (2012) compared type 2 DM patients with no or minimal DR with controls and found that the RNFL, GCL and IPL of the pericentral macula were thinner in the study group. In the study conducted by Shah et al. (2024), layer-specific thinning in diabetic eyes without retinopathy was demonstrated using spectral-domain OCT (SD-OCT) with magnification correction based on axial length. The study revealed that thinning was more prominent in the outer region in eyes with diabetes but no DR, and in the inner region in eyes with mild DR. Detailed analysis showed that GCIPL thinning in the DM no DR group was primarily attributable to the ganglion cell layer (GCL), whereas in the mild DR group, the inner plexiform layer (IPL) was more affected. In our study, we found a statistically significant decrease only in the inferior and inferonasal GCIPL. Although GCC is affected by hyperglycaemia in a multifactorial manner, the neurodegenerative and vascular pathways are generally emphasised. In the neurodegenerative pathway, there may be a slow thinning of the GCC due to atrophy or reduction of neurons. We think that our results may be related with this situation. In the vascular pathway, hyperglycaemia-related microvascular abnormalities lead to increased vascular permeability and capillary dilution, causing intracellular and extracellular oedema in the GCC. Therefore, the thickening of the GCC may be an early sign of the development of macular oedema with vascular leakage (Zeng et al., 2019). However, we found no significant increase in GCC thickness in the study group. We think that this is related to the fact that DR has not yet developed in patients with DPN in the study group and therefore vascular involvement has not yet started.

Dehghani et al. (2017) found that the mean peripapillary RNFL thickness of DPN patients decreased by an average of 0.7 μm per year compared to diabetic patients without DPN. However, Liu et al. (2021) showed increased peripapillary RNFL in both the superior and inferior quadrants in DPN patients. On the other hand, Shahidi et al. (2012) reported in 2012 that RNFL thinning in the lower quadrant was associated with peripheral neuropathy in patients with type 2 DM and was more pronounced in those with a higher risk of foot ulceration. However, we did not find a statistically significant decrease in mean RNFL in the study group in our study.

Our study has several limitations in different aspects. Some of these limitations include the small number of patients, the single-eye study, the absence of HgA1C and hyperlipidaemia and the lack of a one-to-one correlation between axial length and refractive error. In addition, the cross-sectional areas analysed in this study do not allow dynamic assessment of choroidal changes with disease progression. The calculation of CVI is based on the assumption that dark pixelated areas on the choroid represent the vascular areas of the choroid and light pixelated areas represent the stromal areas. However, there are no histopathological studies to confirm this assumption. In a clinical situation that may affect the entire choroidal and retinal vasculature, we were only able to perform a single two-dimensional (2D) scan of a single representative area of 1500 µm to assess vascular changes in the choroid, which may introduce error and limit our results. A complete demographic match (100%) between male participants and the total patient population across study groups was not achieved, indicating slight imbalances in gender distribution.

In conclusion, the present study compared patients with DPN without DR to healthy controls. The results obtained demonstrated a statistically significant increase in LA and TCA in the study group, although CVI remained constant. These two parameters can be used as alternative markers in patients with DPN before the development of DR. Furthermore, a statistically significant decrease in GCIPL thickness in the inferior and inferonasal sectors of the study group was identified, suggesting that GCIPL may serve as a novel non-invasive marker to assess the severity of the disease in patients with DPN without DR. Prospective longitudinal studies are warranted to validate GCIPL thinning and changes in LA/TCA as early predictive biomarkers for the progression of diabetic retinopathy.

## Supplemental Information

10.7717/peerj.20662/supp-1 Supplemental Information 1 Data. In the gender section 1:male, 2: female
In the HT, HT (-) section 1: patients using antihypertensive drugs, 2: Patients not taking antihypertensive medication

10.7717/peerj.20662/supp-2 Supplemental Information 2 Statistical results.

## Additional Information and Declarations

### Data Availability

The following information was supplied regarding data availability:

The raw data is available in the Supplemental File.
